# Tachyplesin Causes Membrane Instability That Kills Multidrug-Resistant Bacteria by Inhibiting the 3-Ketoacyl Carrier Protein Reductase FabG

**DOI:** 10.3389/fmicb.2018.00825

**Published:** 2018-05-01

**Authors:** Cunbao Liu, Jialong Qi, Bin Shan, Yanbing Ma

**Affiliations:** ^1^Laboratory of Molecular Immunology, Institute of Medical Biology, Chinese Academy of Medical Sciences and Peking Union Medical College, Kunming, China; ^2^Department of Clinical Laboratory, The First Affiliated Hospital of Kunming Medical University, Kunming, China

**Keywords:** tachyplesin, FabG, unsaturated fatty acids, membrane rupture, multidrug-resistant bacteria, cytotoxicity, hemolysis

## Abstract

Tachyplesin is a type of cationic β-hairpin antimicrobial peptide discovered in horseshoe crab approximately 30 years ago that is well known for both its potential antimicrobial activities against multidrug-resistant bacteria and its cytotoxicity to mammalian cells. Though its physical interactions with artificial membranes have been well studied, details of its physiological mechanism of action the physiological consequences of its action remain limited. By using the DNA-binding fluorescent dye propidium iodide to monitor membrane integrity, confocal microscopy to assess the intracellular location of FITC-tagged tachyplesin, and RNA sequencing of the differentially expressed genes in four Gram-negative bacteria (*Escherichia coli, Acinetobacter baumannii, Klebsiella pneumoniae*, and *Pseudomonas aeruginosa*) treated with lethal or sublethal concentrations of tachyplesin, we found that compared with levofloxacin-treated bacteria, tachyplesin-treated bacteria showed significant effects on the pathways underlying unsaturated fatty acid biosynthesis. Notably, RNA levels of the conserved and essential 3-ketoacyl carrier protein reductase in this pathway (gene *FabG*) were elevated in all of the four bacteria after tachyplesin treatment. *In vitro* tests including surface plasmon resonance and enzyme activity assays showed that tachyplesin could bind and inhibit 3-ketoacyl carrier protein reductase, which was consistent with molecular docking prediction results. As unsaturated fatty acids are important for membrane fluidity, our results provided one possible mechanism to explain how tachyplesin kills bacteria and causes cytotoxicity by targeting membranes, which may be helpful for designing more specific and safer antibiotics based on the function of tachyplesin.

## Introduction

Tachyplesin is a type of cationic β-hairpin antimicrobial peptide (AMP) discovered from horseshoe crab hemocytes approximately 30 years ago (Nakamura et al., 1988; Miyata et al., 1989; Kawano et al., 1990; Muta et al., 1990). Though it shows broad-spectrum and potent antimicrobial activity on pathogens, especially multidrug-resistant (MDR) pathogens, clinical application of tachyplesin is precluded by its highly hemolytic effects (Ramamoorthy et al., 2006; Cirioni et al., 2007; Liu et al., 2015; Edwards et al., 2017). More ominously, experimental induction of resistance to tachyplesin was reported due to increased proteolytic activity in *Pseudomonas aeruginosa* (Hong et al., 2016). A deep understanding of the unique mechanism of action by which tachyplesin kills bacteria may be helpful to overcome its shortcomings, including hemolysis and susceptibility to proteolysis, and may be helpful for the development of novel antibiotics.

Two models trying to explain how tachyplesin works emerged shortly after it was discovered. One proposed that tachyplesin interacted with lipid membranes and induced enhanced permeability of bacterial membranes, which may contribute to the death of the bacteria (Matsuzaki et al., 1991, 1993; Ohta et al., 1992; Katsu et al., 1993). The other proposed that the antiparallel beta-sheet structure formed by disulfides helped with tachyplesin binding to DNA and might play roles in bacteria killing (Yonezawa et al., 1992). Though the former model seems to be more prevalent, reports concerning this issue are mainly depictions of the physical interactions between this peptide and artificial lipid bilayers, and more biological details are still needed (Doherty et al., 2006, 2008). In any case, two conclusions can be drawn from these observations. The first is that compared with membrane rupture activities, the membrane translocation activity of tachyplesin is more relevant to its antimicrobial potential, which is demonstrated by the fact that linear analogs of tachyplesin without disulfide formation could cause more serious membrane disruptions than a cyclic peptide, which also formed pores during the translocation process but showed weaker membrane translocation ability and weaker antimicrobial activity (Matsuzaki et al., 1993, 1997; Doherty et al., 2006, 2008). The other conclusion is that membrane translocation activity is necessary but not sufficient for tachyplesin to kill bacteria, which is based on the reports that compared to tachyplesin alone, poly(ethylene glycol) (PEG)-grafted (PEGylated) tachyplesin showed similar membrane translocation activity but significantly weakened antimicrobial activity (Imura et al., 2007; Han and Lee, 2013). These phenomena imply that tachyplesin may play an intracellular role.

These speculations have been gradually strengthened by several recent reports. Research using confocal microscopy and flow cytometry to investigate the anti-tumor activities of tachyplesin showed that it induced controlled cell death by promoting apoptosis of the human erythroleukemia cell line K562 at lower concentrations and resulted in cell membrane disruption at higher concentrations (Paredes-Gamero et al., 2012). Proteomic profiling of the effects of tachyplesin on glioblastoma multiforme cell line U251 also implied that tachyplesin may cause cell death by affecting intracellular enzymes and by promoting apoptosis (Li et al., 2017). Regarding its antimicrobial effects, confocal microscopy and flow cytometry showed that bacteria died gradually in response to a sublethal concentration of tachyplesin and died instantaneously upon exposure to high concentrations of tachyplesin. Though this process was accompanied by the inactivation of intracellular esterases, no further details were reported (Hong et al., 2015).

A systemic view of how bacteria react to antibiotics by omics assay may reflect pathways with which these antibiotics interfere and is helpful to elucidate undefined mechanisms of novel antibiotics, including AMPs (Kohanski et al., 2010; Tan et al., 2014; Ho et al., 2016; Pulido et al., 2016; Shah et al., 2016). In the present study, we treated MDR clinically isolated pathogens with lethal and sublethal doses of tachyplesin, tested the membrane translocation activity of fluorescein isothiocyanate (FITC)-tagged tachyplesin with confocal microscopy, assessed the membrane rupture activity using DNA-binding fluorescent dye propidium iodide (PI), and further analyzed the differentially expressed RNA-sequencing data with standard bioinformatics analyses, such as Kyoto Encyclopedia of Genes and Genomes (KEGG) analysis. *In vitro* tests, including surface plasmon resonance and enzyme activity assays, molecular docking predictions, and RNA interference, were also performed to confirm the potential targets of tachyplesin identified by RNA sequencing data.

## Materials and Methods

### Ethics Statement

The animal experimental procedures were approved by the Ethics Committee of Animal Care and Welfare of the Institute of Medical Biology, Chinese Academy of Medical Sciences (CAMS) and Peking Union Medial College (PUMC) (Permit Number: SYXK (dian) 2010-0007). All efforts were made to minimize animal suffering.

### Serum Stability, Cytotoxicity, Membrane Rupture and Translocation Activity

These studies were carried out as we previously reported with slight modifications and briefly introduced as follows (Liu et al., 2017). Serum stability was assessed with increases over the minimal inhibitory concentration (MIC) of tachyplesin III (KW*C*FRVCYRGICYRK*C*R; cysteines with the same type font formed on disulfide; purity ≥ 95%, synthesized by GL Biochem Co., Ltd, Shanghai, China) on *Escherichia coli* strain DH5α after incubation in serum. Cytotoxicity in different cell lines was assessed using the Cell Proliferation Kit II (XTT) (Roche), and cytotoxicity in hemocytes was evaluated by the increase in the absorbance at 540 nm after peptide incubation. Membrane rupture was indicated by the entrance of the DNA-binding fluorescent dye PI into MDR clinical isolates of *P. aeruginosa* strain 1409. Membrane translocation of N-terminal FITC-conjugated tachyplesin III (purity ≥ 95%, synthesized by GL Biochem Co., Ltd, Shanghai, China) into MDR clinical isolates of *Acinetobacter baumannii* strain 1408 were detected by confocal laser-scanning microscopy.

### Transcriptome Analysis

The Vitek 32 system (bioMerieux, France) and 16S rDNA sequencing were used to verify the clinical isolates *A. baumannii* 1408, *P. aeruginosa* 1409, *Klebsiella pneumoniae* 5 and *E. coli* 513, which were grown overnight in Luria-Bertani (LB) medium at 37°C with constant shaking at 220 rpm to reach the middle of their logarithmic growth phase. A final concentration of 16 μg/ml tachyplesin III (1×, 2×, 1/2×, and 4× MICs for *A. baumannii* 1408, *P. aeruginosa* 1409, *K. pneumoniae* 5 and *E. coli* 513, respectively) and 32 μg/ml levofloxacin (1×, 2×, 2×, and 1× MICs for *A. baumannii* 1408, *P. aeruginosa* 1409, *K. pneumoniae* 5, and *E. coli* 513, respectively) were added, and the cultures were incubated at 37°C for 30 min. After the addition of 2 volumes of RNAprotect Bacteria Reagent (QIAGEN) to stabilized RNA, samples were vortexed for 5 s, incubated for 5 min at room temperature (15–25°C) and collected by centrifugation at 4°C for 5 min at 4000 *g*.

RNA isolation and sequencing were performed by Novogene Bioinformatics Technology Co., Ltd. (Beijing, China) as previously described (Liu B. et al., 2014). Clean reads were mapped to the corresponding bacterial genomes using Bowtie 2 software (Langmead and Salzberg, 2012). Gene expression was calculated using the FPKM (expected number of fragments per kilobase of transcript sequence per millions of base pairs sequenced) method with HTSeq software (union model) (Anders et al., 2015). Differentially expressed genes were selected based on the DESeq R package with Padj values < 0.05 (Anders and Huber, 2010). KEGG pathway analysis was performed using KOBAS (2.0), and the corrected *p* value cut-off was set at 0.05 (Li et al., 2015).

### Verification of *FabG* Transcription Levels by Quantitative Real-Time PCR

Quantitative real-time PCR (RT-PCR) was performed following the 2^-ΔΔCT^ method with RNAs from the aforementioned transcriptome analysis (Livak and Schmittgen, 2001; Liu C. et al., 2014). Untreated pathogen strains were used as the calibrator strains, and corresponding ribosomal *rpsL* genes were used as housekeeping reference genes with the primers shown in Supplementary Table S1 (Dumas et al., 2006).

### RNA Interference (RNAi)

*Pseudomonas aeruginosa* 1409 incubated overnight in LB medium at 37°C were re-incubated under the same conditions to reach an OD600 of 0.5–1.0, put on ice for 15 min to stop growth, washed three times with sterile deionized water and collected by centrifugation at 4°C for 15 min at 4000 × *g*. After washing with sterile 10% glycerol in water (v/v), cells were collected via the aforementioned centrifugation conditions and re-suspended in 10% glycerol in water (v/v) to obtain electrotransformation-competent *P. aeruginosa* cells.

With the RNA oligo pairs shown in Supplementary Table S2 (GenePharma Co., Ltd, Shanghai, China) targeting *FabG* from *P. aeruginosa*, RNAi was carried out with a GenePulser Xcell system using a 2 mm cuvette with the default settings for *P. aeruginosa* (2.5 kV/cm, 25 μF and 200 Ω). The transformants were either spread onto LB agar plates to calculate the number of bacterial clones or incubated in LB medium at 37°C for 40 min to determine *FabG* transcript levels by RT-PCR using the primers for *P. aeruginosa* shown in Supplementary Table S1.

### Analysis of Binding Interaction Between Tachyplesin and FabG Protein

N-terminal His-tagged FabG from *E. coli* and C-terminal His-tagged FabG from *Staphylococcus aureus* were cloned into the NcoI and XhoI cloning sites of the vector pET28a, expressed in BL21 DE3 *E. coli* and further purified with Ni resin (Kristan et al., 2009; Srinivas and Cronan, 2017). The binding interaction between tachyplesin III and FabG was analyzed by surface plasmon resonance (SPR) with a Biocore 3000 (Biocore, Piscataway, NJ, United States). Briefly, tachyplesin III was adsorbed onto a CM5 sensor chip using an amine-coupling kit (GE Healthcare, Little Chalfont, United Kingdom) to obtain approximately 1000 resonance units. For the analysis, different concentrations of FabG proteins in running buffer (HBS-N buffer, GE Healthcare) were injected at a flow rate of 20 μl/min for 6 min. The binding affinity of FabG for tachyplesin III was determined by BIAevaluation 3.0 software (Biacore) with a 1:1 Langmuir binding model for the kinetic calculation (Wei et al., 2013).

### FabG Enzyme Activity Inhibition Assay

A 96-well plate with a final total volume of 200 μl per reaction was used for the FabG inhibition assay. Briefly, tachyplesin III (15, 7.5, and 3.75 μM) was incubated with 1.5 μM FabG from *E. coli* or 6 μM FabG from *S. aureus* at room temperature for 5 min, before the addition of 300 μM nicotinamide adenine dinucleotide phosphate (NADPH, Sigma) and then 150 μM acetoacetyl-CoA (Sigma) to initiate the reaction. The decrease in absorbance at 340 nm was recorded over 5 min to reflect the consumption of NADPH.

### Molecular Docking Prediction

Tachyplesin III was processed with Chemdraw 11.0 according to the structure of tachyplesin I (PDB code 2RTV) and docked to the active site of FabG from *E. coli* (PDB code 1Q7B) or *Staphylococcus aureus* (PDB code 3SJ7) with AutoDock4 using the default settings (Price et al., 2004; Morris et al., 2009; Dutta et al., 2012; Kushibiki et al., 2014).

## Results

### Tachyplesin III Was Stable in Mouse Serum

**FIGURE 1:**
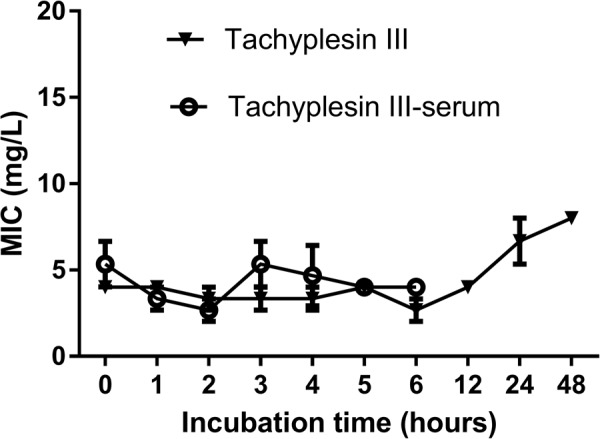
Serum stability of tachyplesin III. Tachyplesin III (2 mg/ml) was incubated in either sterile deionized water or mouse serum at 37°C for various lengths of time, aliquots were taken, and MICs against DH5α cells were tested. Stability of the MIC indicates the stability of tachyplesin III.

Tachyplesin III was stable after incubation in sterile deionized water at 37°C for nearly 48 h, reflected by the fact that the MIC against DH5α increased slightly from 4 mg/L to 8 mg/L after 24 h of incubation. A similar phenomenon was observed when tachyplesin III was incubated with mouse serum, which was tested during the first 6 h (**Figure 1**).

### Tachyplesin Was Cytotoxic to Mammalian Cells at High Concentrations

**FIGURE 2:**
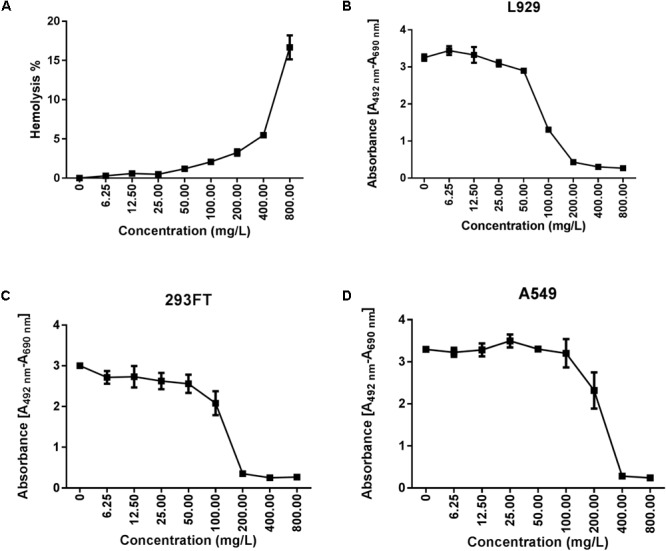
Cytotoxicity of tachyplesin III. Cytotoxicity of tachyplesin III was tested by hemolysis and cell viability assays. **(A)** Tachyplesin III dissolved in 0.9% saline was added to mouse erythrocytes that were diluted with the same buffer. After 30 min of incubation at 37°C and then centrifugation at 1000 *g* for 15 min, supernatants were diluted 1:4 with 0.9% saline, and absorbance at 540 nm was tested. With 1% Triton X-100 (v/v) to determine 100% hemolysis and 0.9% saline as the negative control, the hemolysis rate was calculated as [(Absorbance_sample_^-^Absorbance_control_)/(Absorbance_100%_^-^Absorbance_control_)] × 100. Cytotoxicity was assessed using the Cell Proliferation Kit II (XTT) (Roche) for **(B)** L929 (mouse fibroblast cell line), **(C)** 293FT (human embryonic kidney cell line) and **(D)** A549 (adenocarcinomic human alveolar basal epithelial cell line) cells. Absorbance [A492-A690 nm] was used to quantify viable cells.

Approximately 1% and 2% hemolysis was observed when tachyplesin III was applied at concentrations of 50 and 100 mg/L, respectively, and hemolysis accelerated as the concentration of tachyplesin increased further (**Figure 2A**). Cell viability of L919 cells, the murine fibroblast cell line used for toxicity testing, declined sharply when the concentration of tachyplesin increased from 50 mg/L to 100 mg/L (**Figure 2B**). Dramatic decreases in viability were observed in 293FT human embryonic kidney cells when tachyplesin was increased from 100 mg/L to 200 mg/L (**Figure 2C**) and in A549 adenocarcinomic human alveolar basal epithelial cells when tachyplesin was increased 200 mg/L to 400 mg/L (**Figure 2D**).

### Effect of Tachyplesin on Pathogens at Low and High Concentrations

**FIGURE 3:**
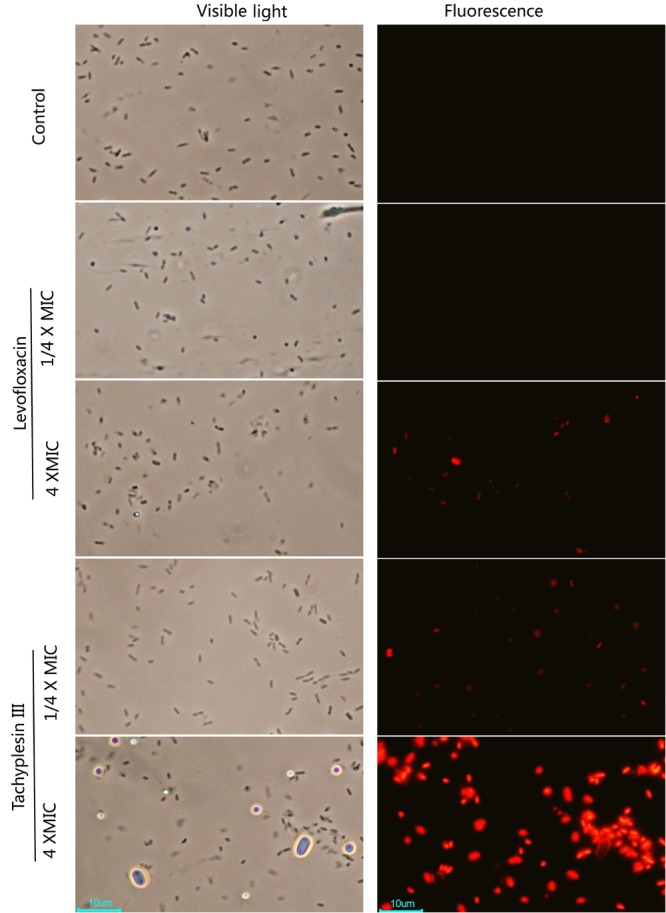
Propidium iodide staining after treatment with levofloxacin or tachyplesin III at different concentrations. First, 1 × 10^7^ MDR clinical isolates of *P. aeruginosa* strain 1409 were incubated with 1/4× MIC (4 μg/ml), 4× MIC (64 μg/ml) levofloxacin (Tokyo Chemical Industry Co. Ltd) or 1/4× MIC (2 μg/ml), 4× MIC (32 μg/ml) tachyplesin III in Luria-Bertani (LB) medium at 25°C for 1 h. After centrifugation at 3000 *g* for 10 min, cells were resuspended in the same volume of PBS (phosphate-buffered saline, 8 g/L NaCl, 0.2 g/L KCl, 1.44 g/L Na_2_HPO_4_, 0.24 g/L KH_2_PO_4_, pH 7.4). Then, PI was added to a final concentration of 10 μg/ml. After 30 min of incubation at 25°C, the cells were washed three times with PBS and immediately imaged with a fluorescence microscope.

PI is a DNA-binding fluorescent dye that can penetrate broken membranes but not intact membranes. As shown in **Figure 3**, while levofloxacin at low concentrations (1/4× MIC) did not affect the integrity of bacterial membranes, some of the bacterial membranes were broken slightly after treatment with levofloxacin at 4× MIC for 1 h at 25°C, as indicated by the emergence of weak red PI fluorescence inside these cells. Interestingly, tachyplesin at low concentrations (1/4× MIC) could also induce slight membrane breakage in a small proportion of the treated bacteria, while tachyplesin at high concentrations induced complete membrane breakage of nearly all of the treated bacteria.

**FIGURE 4:**
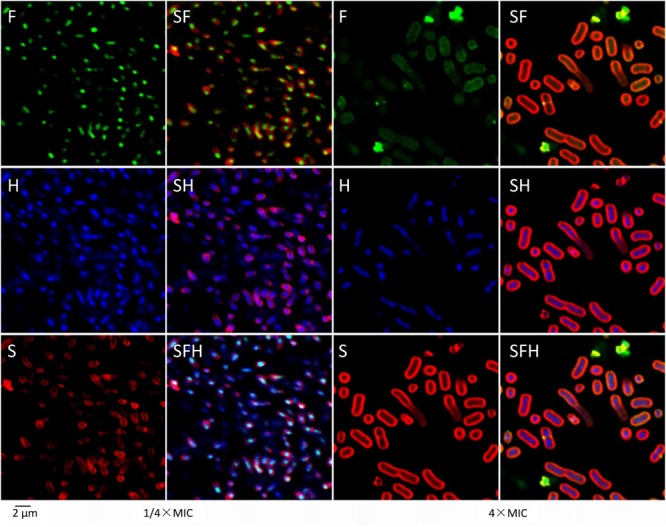
Localization of tachyplesin III with confocal laser-scanning microscopy. First, 1 × 10^7^ MDR clinical isolates of *A. baumannii* strain 1408 were incubated with 1/4× MIC (2 μg/ml) or 4× MIC (32 μg/ml) N-terminally tagged tachyplesin III in LB medium at 25°C for 1 h. After centrifugation at 3000 *g* for 10 min, cells were resuspended with the same volume PBS and incubated with 20 μg/ml Hoechst (Sigma) at room temperature for 20 min. After another centrifugation at 3000 *g* for 10 min, cells were resuspended with the same volume of Hank’s solution (8 g/L NaCl, 0.4 g/L KCl, 1 g/L glucose, 60 mg/L KH_2_PO_4_, 47.5 mg/L Na_2_HPO_4_, pH 7.2) and incubated with 20 μg/ml SynaptoRed C2 (Tocris Bioscience) on ice for 1 min. Confocal laser-scanning microscopy was performed with excitation and emission wavelengths, respectively, of 488 nm and 530 nm for FITC, 352 nm and 461 nm for Hoechst, and 515 and 640 nm for SynaptoRed C2.

Confocal laser-scanning microscopy showed more detailed results (**Figure 4**). After treatment with 1/4× MIC at 25°C for 1 h, most of the FITC-tagged tachyplesin (green in **Figure 4F**, left) co-localized with DNA (stained blue by Hoechst in **Figure 4H**, left) rather than with bacterial membranes (stained red by SynaptoRed C2 in **Figure 4S**, left). Treatment with tachyplesin at 4× MIC showed completely different results: tachyplesin was dispersed all over the bacteria (**Figure 4F**, right), including nuclear regions (**Figure 4H**, right) and bacterial membranes (**Figure 4S**, right). Notably, bacteria treated with tachyplesin at 4× MIC showed marked shape changes, including elongated nuclear regions (**Figure 4H**, right vs. **Figure 4H**, left) and enlarged cell bodies (**Figure 4S** right vs. **Figure 4S** left).

### Pathways for the Biosynthesis of Unsaturated Fatty Acids Were Altered in All of the Pathogens Treated With Tachyplesin but Not Levofloxacin

The raw sequence data reported in this paper have been deposited in the Genome Sequence Archive at the Beijing Institute of Genomics, Chinese Academy of Sciences, under accession number CRA000801 and are publicly accessible at http://bigd.big.ac.cn/gsa (Wang et al., 2017; Big Data Center Members, 2018). Unique read numbers for each expressed gene were calculated using FPKM and are shown in Supplementary Data Sheet S1. Fold changes of expressed genes and corresponding *p*-values are shown in Supplementary Data Sheet S2.

Among all of the KEGG pathways shared in all of the pathogens subjected to transcriptional analysis after tachyplesin treatment, the “biosynthesis of unsaturated fatty acids” pathway (KEGG ID 01040) was not enriched in any of the pathogens treated with levofloxacin (**Table 1**). Although the geraniol degradation pathway (KEGG ID 00281) was detected in the transcriptional analysis of tachyplesin-treated pathogens and undetected in levofloxacin-treated pathogens, such transcriptional changes were not shared by all of the pathogens tested, and this pathway was excluded from further analysis. The nicotinate and nicotinamide metabolism pathway, which was shared among all of the pathogens subjected to transcriptional analysis (and verified on the proteomic level based on unpublished data) after tachyplesin treatment, was excluded for similar reasons. Though transcriptional changes in this pathway were not detected after levofloxacin treatment, this pathway was affected at the proteomic level after levofloxacin treatment in both of the pathogens tested (*A. baumannii* 1408 and *P. aeruginosa* 1409, in unpublished data).

**Table 1:** Affected KEGG pathways shared among Ta-treated pathogens after transcriptome analysis and their existence in Lvf-treated pathogens.

KEGG ID	Terms shared	*P*_min_	Detected in Lvf-treated pathogens
00281	Geraniol degradation (Kp-)^a^	0.000142	–
00280	Valine, leucine, and isoleucine degradation	0.005811	+
00071	Fatty acid degradation	0.0099055	+
01212	Fatty acid metabolism	0.0045136	+
01040	Biosynthesis of unsaturated fatty acids	0.070313	–
00380	Tryptophan metabolism	0.0868888	+
00362	Benzoate degradation	0.0951633	+
00310	Lysine degradation	0.1114944	+
00410	beta-Alanine metabolism (Ab-)^b^	0.135456	+
00760	Nicotinate and nicotinamide metabolism	0.0011823	–
00230	Purine metabolism	0.1948386	+
00270	Cysteine and methionine metabolism (Ab-)^b^	0.0055591	+
00640	Propanoate metabolism	0.083494	+
00650	Butanoate metabolism	0.128707	+
01110	Biosynthesis of secondary metabolites	0.1493282	+
00240	Pyrimidine metabolism (Ab-)^b^	0.4485617	+
01200	Carbon metabolism	0.1854894	+
01230	Biosynthesis of amino acids (Ab-)^b^	0.55726	+
01120	Microbial metabolism in diverse environments	0.0306	+
01100	Metabolic pathways	0.27439	+
02020	Two-component system	0.04481	+

*KEGG, Kyoto Encyclopedia of Genes and Genomes; Pmin, minimum *P*-value detected; Ta, tachyplesin III; Lvf, levofloxacin; Ab, *A. baumannii*; Kp, K. *pneumoniae*. -, undetected; +, detected; ^*a*^undetected in *K. pneumoniae*; ^*b*^undetected in *A. baumannii*.*

### Expression of *FabG*, Part of the Unsaturated Fatty Acid Biosynthetic Pathway, Was Elevated After Tachyplesin Treatment in All Pathogens Tested

**FIGURE 5:**
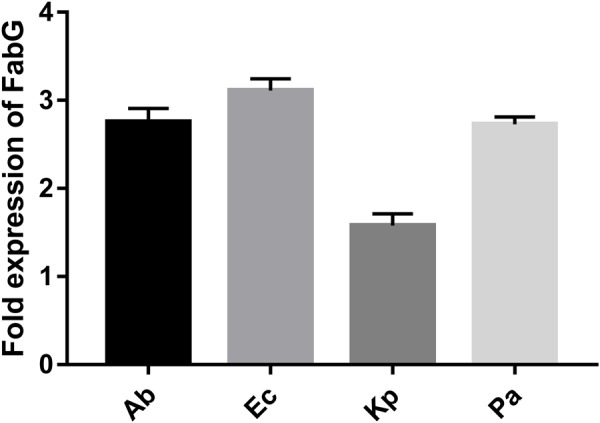
Expression of *FabG* increased after tachyplesin treatment. Quantitative real-time PCR was performed following the 2^-ΔΔCT^ method. Untreated pathogen strains were used as the calibrator strains, and the corresponding ribosomal *rpsL* genes were used as housekeeping reference gene with the primers shown in Supplementary Table S1.

Supplementary Figure S1 shows details of the biosynthetic pathways for unsaturated fatty acids of all the bacteria tested. Notably, the *FabG* gene, which encodes 3-ketoacyl-acyl carrier protein reductase, was the only gene with elevated expression detected after tachyplesin III treatment in all four bacteria (**Table 2**). These elevations after tachyplesin III treatment were also confirmed by quantitative real-time PCR (**Figure 5**).

**Table 2:** Differentially expressed genes in the biosynthesis of unsaturated fatty acids pathways after treatment.

Bacteria	Gene ID	FPKM			Fold change after Ta treatment	*P* value after Ta treatment	Description
		Control	Lvf	Ta			
Ab	A1S_2061	323	328	891	2.89	7.42E-79	3-ketoacyl-acyl carrier protein reductase
Ec	b1093 b3846	633 97	627 94	715 260	1.11 2.3	0.057 9.78E-39	3-ketoacyl-acyl carrier protein reductase 3-hydroxyacyl-CoA dehydrogenase
Kp	KPNJ2_03426 KPNJ2_01584	41.4 128	80.6 190	69 237	1.83 2	0.02 3.04E-17	3-ketoacyl-acyl carrier protein reductase Enoyl-CoA hydratase
Pa	PADK2_25405 PADK2_09275	56 361	43 369	160 774	2.71 2.04	4.51E-17 5.71E-74	3-ketoacyl-acyl carrier protein reductase 3-hydroxyacyl-CoA dehydrogenase

*Ab, *A. baumannii*; Ec, *E. coli*; Kp, *K. pneumoniae*; Pa, *P. aeruginosa*; FPKM, expected number of fragments per kilobase of transcript sequence per millions of base pairs sequenced; Ta, tachyplesin III; Lvf, levofloxacin.*

### RNA-Silencing of *FabG* Reduced Bacterial Colony Counts

**FIGURE 6:**
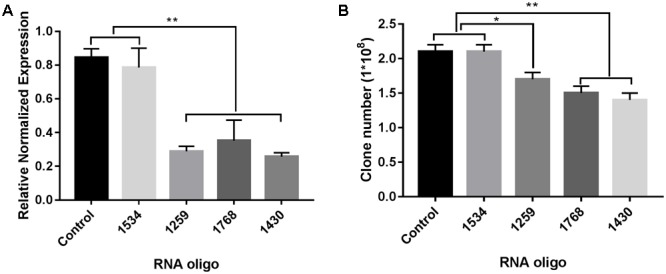
RNA silencing of *FabG* reduced bacterial colony counts. Using the RNA oligo pairs shown in Supplementary Table S2 (GenePharma Co., Ltd, Shanghai, China) to target *FabG* from *P. aeruginosa*, RNAi was carried out with a GenePulser Xcell system using a 2 mm cuvette with the default settings for electrotransformation-competent *P. aeruginosa* cells (2.5 kV/cm, 25 μF and 200 Ω). The transformants were either spread onto LB agar plates to calculate bacterial clones **(B)** or incubated in LB medium at 37°C for 40 min to determine *FabG* transcripts by RT-PCR **(A)** with the primers for *P. aeruginosa* shown in Supplementary Table S1. Statistical significance was analyzed by one-way ANOVA followed by the Newman–Keuls multiple-comparisons test, ^∗∗^*p* < 0.01, ^∗^*p* < 0.05.

As shown in **Figure 6A**, three of the RNA oligos (1259, 1768, and 1430) that we selected worked well and significantly down-regulated the expression of *FabG* in *P. aeruginosa* (*p* < 0.01), which was accompanied by reduced bacterial colony survival (**Figure 6B**). However, for RNA oligo 1534, which was inefficient at down-regulating *FabG* in *P. aeruginosa*, transformation of this RNA oligo had no effect on the survival rate of bacterial colonies.

### Tachyplesin III Binding With FabG Inhibited Its Enzyme Activities *in Vitro*, Likely by Blocking the Active Site

**FIGURE 7:**
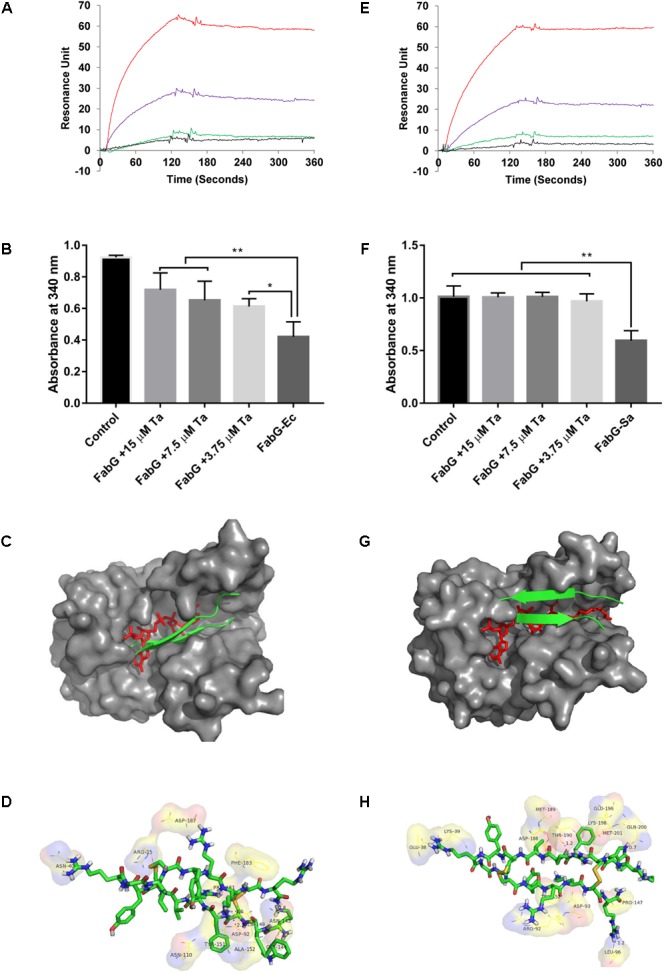
Tachyplesin III binding with FabG inhibits its enzyme activities *in vitro*, probably by blocking the active site. Binding interactions between tachyplesin III and FabG from *E. coli* **(A)** or from *S. aureus* **(E)** were analyzed by surface plasmon resonance (SPR) with a Biocore 3000 (Biocore, Piscataway, NJ, United States). Black line: 1.25 μg/mL FabG; green line: 2.5 μg/mL FabG; purple line: 5 μg/mL FabG; and red line: 10 μg/mL FabG. The inhibitory activity of tachyplesin III on FabG from *E. coli* **(B)** or from *S. aureus* **(F)** was evaluated based on the remaining NADPH (which shows absorbance at 340 nm at 𝜀_340_ = 6.3 × 10^3^ mol^-1^) after FabG consumption for 5 min at room temperature. Tachyplesin was processed with Chemdraw 11.0 according to the structure of tachyplesin I (PDB code 2RTV) and then docked to the active site of FabG from *E. coli* (PDB code 1Q7B) or *S. aureus* (PDB code 3SJ7) with AutoDock4 using the default settings. Docking of tachyplesin III into *E. coli* is shown in **(C)**, docking details are shown in **(D)**. Docking of tachyplesin III into *S. aureus* is shown in **(G)**, docking details are shown in **(H)**. In **(C,G)**, tachyplesin III is shown in green, and NADPH is shown in red. Statistical significance was analyzed by one-way ANOVA followed by the Newman–Keuls multiple-comparisons test, ^∗∗^*p* < 0.01, ^∗^*p* < 0.05.

Surface plasmon resonance analysis showed that tachyplesin III could bind with FabG from *E. coli* (**Figure 7A**) or *S. aureus* (**Figure 7E**) in a dose-dependent manner. This binding interaction between tachyplesin III and FabG is accompanied by inhibition of the enzyme activities of FabG (**Figures 7B,F**). While FabG from *E. coli* consumed 54% of NADPH after incubation for 5 min at room temperature, incubation with 15, 7.5 and 3.75 μM tachyplesin III reduced the consumption rates to 22, 29, and 34%, respectively (**Figure 7B**). The tests with FabG from *S. aureus* showed even more marked results. While FabG from *S. aureus* consumed 42% of NADPH after 5 min of incubation at room temperature, only 4% of NADPH was consumed after incubation with 3.75 μM tachyplesin III, and FabG activity was almost completely blocked after incubation with higher concentrations of tachyplesin III (**Figure 7F**).

As tachyplesin III could bind with FabG and inhibit its enzyme activities, we next predicted whether tachyplesin III could block the active site of FabG. Molecular docking analysis results showed that tachyplesin III could fit into the substrate-binding region of FabG proteins from both *E. coli* (**Figure 7C**) and *S. aureus* (**Figure 7G**), which is consistent with its efficacy against both Gram-negative and Gram-positive bacteria. Detailed analysis showed that tachyplesin could interact with the active site of FabG via hydrophobic interactions and van der Waals forces. Tachyplesin interacted with R15, N145, Y151, A152, N40, D92, and F183 and formed hydrogen bonds with N40, D92, N145, and Q148 in the active site of *E. coli* FabG (**Figure 7D**); in *S. aureus*, it interacted with K39, R92, D93, L96, P147, Y152, K198, and Q200 and formed hydrogen bonds with Leu96, Thr190, and Gln200 in the active site (**Figure 7H**).

## Discussion

Since the discovery of tachyplesin approximately 30 years ago, its potential and broad-spectrum activities against MDR bacteria have been repeatedly confirmed (Nakamura et al., 1988). Though experimental induction of resistance to tachyplesin was reported recently in Gram-negative bacteria, with the resistance mechanism of *P. aeruginosa* “not entirely dependent on extracellular proteolytic degradation of tachyplesin,” this resistance ability quickly disappeared after 6–9 passages without tachyplesin pressure because maintaining resistance may be a huge cost, similar to resistance to colistin (Fernandez-Reyes et al., 2009; Hong et al., 2015). Moreover, our results showed that tachyplesin III was stable in mouse serum for at least 6 h (**Figure 1**). As mouse serum is enriched in endogenous mammalian proteases, these results demonstrated that tachyplesin was resistant to proteolysis and may be stable *in vivo*.

Compared with serum stability issues, cytotoxicity seems to be the real inherent defect hindering the clinical application of tachyplesin (Edwards et al., 2016). At a final concentration of 100 mg/L, tachyplesin caused lysis of approximately 2% of hemocytes (**Figure 2A**), as well as a decline in the cell viability of 293FT human embryonic kidney cells (**Figure 2C**) and a sharp decline in the cell viability of L919 murine fibroblasts (**Figure 2B**), a cell line commonly used for toxicity testing. Interestingly, tumor cells such as A549 adenocarcinomic human alveolar basal epithelial cells seem to be more resistant to tachyplesin (**Figure 2D**), which made the reports of its anticancer effects somewhat controversial (Evans et al., 2017; Li et al., 2017).

A deep understanding of how tachyplesin works may help in developing novel effective antibiotics based on similar mechanisms while avoiding the problematic cytotoxicity. Compared with the DNA binding theory, the membrane rupture theory seems to be more likely as bacterial membrane rupture is an obvious phenomenon after tachyplesin treatment at both lethal and sublethal concentrations (**Figure 3**). It is natural to deduce that tachyplesin may act directly on membranes and form leakage pores to kill bacteria, and numerous studies have focused on the direct interactions between tachyplesin and membranes, mainly using artificial lipid bilayers (Matsuzaki et al., 1997; Doherty et al., 2006; Han and Lee, 2015). Interestingly, these studies have found that linear analogs of tachyplesin without disulfides also possess the membrane disruptive abilities of original tachyplesin but translocate through these bilayers inefficiently and accompanied by weaker antibacterial activities, implying that membrane translocation abilities instead of direct membrane rupture abilities are vital for its antibacterial activity (Matsuzaki et al., 1997). Furthermore, PEGylation of tachyplesin did not alter the toroidal pore formation and membrane translocation activities of tachyplesin but reduced its cytotoxicity and antimicrobial activities, implying that events inside the cell may determine both the cytotoxicity and the antimicrobial activities of tachyplesin (Imura et al., 2007).

Proteomic profiling showed that low concentrations of tachyplesin may promote apoptosis in the glioblastoma multiforme cell line U251 by affecting intracellular enzymes and cause cell death in *E. coli* by inactivating intracellular esterases (Hong et al., 2015; Li et al., 2017). However, treatment with a high concentration of tachyplesin caused the rapid death of K562 human erythroleukemia cells and *E. coli*, accompanied by complete cell membrane rupture in both cases (Paredes-Gamero et al., 2012; Hong et al., 2015). This phenomenon was also observed in our studies, in which sublethal concentrations of tachyplesin aggregated at specific regions within bacteria (**Figure 4**, 1/4× MIC) and caused slight membrane rupture in a small proportion of the bacteria (**Figure 3**, 1/4× MIC), whereas a lethal concentration of tachyplesin dispersed all over the bacteria (**Figure 4**, 4× MIC) and caused complete membrane rupture in nearly all of the treated bacteria (**Figure 3**, 4× MIC).

Two phenomena should be noted among these results. First, only some of the bacteria treated with lethal concentrations of levofloxacin showed membrane rupture, and the membrane rupture was minor in most cases, as demonstrated by the limited entrance of PI into treated bacteria and the weak fluorescent signals detected (**Figure 3**, 4× MIC for levofloxacin). As levofloxacin is known to disturb DNA separation and supercoiling by inhibiting bacterial topoisomerases, thereby killing the cells, the slight membrane rupture is presumably the result of cell death (Drlica and Zhao, 1997; Ferrandiz and de la Campa, 2014). By contrast, nearly all the bacteria treated with lethal concentrations of tachyplesin showed bright fluorescent signals (**Figure 3**, 4× MIC for tachyplesin), demonstrating that all of the bacteria killed by tachyplesin showed thorough membrane rupture and thus indicating that tachyplesin likely kills bacteria by targeting the membrane. To our surprise, existing theories concerning this topic are mainly based on the depiction of the physical interactions between this peptide and artificial lipid bilayers, and few biological details are offered. A dual effect theory emerged recently, suggesting that tachyplesin enter bacteria via the formation of non-lethal membrane pores, cause sub-lethal effects at low concentrations by inactivating intracellular enzymes, and cause cell membrane disruption at high concentrations. It may be difficult for a peptide to perform so many functions to kill bacteria, and some of those functions are unnecessary because they are not lethal. Moreover, no further biological details concerning membrane rupture have been reported (Paredes-Gamero et al., 2012; Hong et al., 2015). Second, bacteria treated with lethal concentrations of tachyplesin showed elongated nuclear regions (**Figure 4H**, right vs. **Figure 4H**, left) and enlarged cell bodies (**Figure 4S**, right vs. **Figure 4S**, left). This phenomenon was revealed by an approach called bacterial cytological profiling (BCP) that is quite helpful for characterizing the action mechanisms of antibiotics (Nonejuie et al., 2013). Though membrane lengths and DNA lengths of bacteria treated with lethal concentrations of tachyplesin were approximately twice those of bacteria treated with sublethal concentrations, which is consistent with the lipid biosynthesis inhibitors reported in this paper, we have no corresponding models or exact parameters for determining the mechanism of action with the BCP method.

As our laboratory could not conduct the BCP analysis, we turned to omics techniques to identify the effects of tachyplesin treatment on bacteria and to elucidate its action mechanisms (Kohanski et al., 2010; Pulido et al., 2016). Compared with levofloxacin-treated bacteria, all of the pathogens treated with tachyplesin III showed marked effects on the biosynthesis of unsaturated fatty acids, which is important for membrane fluidity, and this result was verified at the protein level (**Table 1**). In this pathway, the β-ketoacyl-acyl carrier protein (ACP) reductase FabG was up-regulated in all the bacteria tested (**Figure 5** and **Table 2**). RNA silencing of *FabG* showed that efficient silencing of *FabG* by RNA oligos (**Figure 6A**) is accompanied by the reduced survival of bacterial colonies (**Figure 6B**), which is consistent with previous reports that *FabG* is a conserved and essential gene in various bacteria such that knockout of this enzyme results in bacterial death (Zhang et al., 2003; Lai and Cronan, 2004; Baba et al., 2006; Parish et al., 2007; Cukier et al., 2013).

The biosynthesis of unsaturated fatty acids occurs in the cytoplasm, and the membrane translocation activities of tachyplesin accompany its antimicrobial activities. *FabG* is essential for the growth of bacteria, and inhibition of FabG is associated with antimicrobial activity in both *E. coli* and *S. aureus* (Sohn et al., 2008). Thus, we propose that tachyplesin may kill bacteria by inhibiting FabG activities. Our surface plasmon resonance analysis results showed that tachyplesin III could bind with FabG from *E. coli* (**Figure 7A**) and *S. aureus* (**Figure 7E**) in a dose-dependent manner. This binding interaction between tachyplesin III and FabG is accompanied by inhibition of the enzyme activities of FabG (**Figures 7B,F**). Molecular docking predictions showed that tachyplesin III could not only enter the active site of FabG from *E. coli* by hydrophobic interactions and van der Waals forces but also block the NADPH binding site by interacting with the corresponding amino acid residues and forming stable hydrogen bonds (**Figures 7C,D**). A similar phenomenon was observed for the interaction of tachyplesin III and Gram-positive *S. aureus* (**Figures 7G,H**), which is consistent with the broad antimicrobial spectrum of tachyplesin.

Many antibiotics targeting bacterial pathways for fatty acid biosynthesis show some cytotoxicity to mammalian cells. For example, triclosan can kill bacteria by binding its enoyl-acyl carrier protein reductase (Nonejuie et al., 2013), but it also shows severe hemolytic activity (Singh et al., 2003). A similar phenomenon was observed for cerulenin, which can kill bacteria by binding bacterial fatty acid synthase (Nonejuie et al., 2013). Though no hemolytic activity was reported, cerulenin was reported to be cytotoxic to carcinoma cells (Pizer et al., 1996). Whether tachyplesin III may cause toxicity to mammalian cells via similar mechanism remains to be validated. Though the fatty acid synthetic pathway in bacteria (FAS II) consists of multiple individual enzymes, whereas the fatty acid synthetic pathway in mammals (FAS I) is a single protein with multifunctional polypeptides, sequence homologies may exist at similar active sites, and inhibitors may have similar effects on both the bacterial and mammalian fatty acid synthesis pathways. These phenomena remind us that more-specific inhibitors may be selected or designed to avoid cytotoxic effects when targeting these fatty acid synthases.

Efficient entrance into cells to act on FabGs that are located in the cytoplasm may be another strategy that we should adopt from tachyplesin. It was reported that inhibitors targeting an allosteric binding site of FabG showed effects only toward Gram-positive bacteria and not toward Gram-negative *P. aeruginosa* (Cukier et al., 2013). This may be attributable either to poor penetration activities of these inhibitors or to rapid efflux by *P. aeruginosa*. Though tachyplesin seems to target a different active site, designing inhibitors based on the action mechanism of tachyplesin and possessing penetration activity may be beneficial.

## Conclusion

We find that tachyplesin may kill bacteria by targeting FabG, the conserved β-ketoacyl-acyl carrier protein reductase in unsaturated fatty acid biosynthesis. Targeting the homologous enzyme in mammalian cells may also result in instability or rupture of cell membranes, which manifested as cytotoxicity, including hemolysis. More-detailed research concerning the structural differences between bacterial and mammalian FabG proteins, as well as the selection of more specific inhibitory ligands, may be helpful for the development of safer antibiotics designed based on the mechanism of tachyplesin.

## Author Contributions

CL conceived and designed the experiments. CL, JQ, and BS performed the experiments. CL and JQ analyzed the data. BS contributed reagents, materials and analysis tools. CL and YM contributed to the writing of the manuscript.

## Conflict of Interest Statement

The authors declare that the research was conducted in the absence of any commercial or financial relationships that could be construed as a potential conflict of interest.
