# Supplementary material for: Tachyplesin Causes Membrane Instability That Kills Multidrug-Resistant Bacteria by Inhibiting the 3-Ketoacyl Carrier Protein Reductase FabG
Source: Front Microbiol. 2018 May 1;9:825. doi: 10.3389/fmicb.2018.00825 (PMC5938390; doi:10.3389/fmicb.2018.00825)
Supplement: Supplementary file 1 [file Table_1.DOCX]

**Table S1 Primers for RT PCR**

| Pathogen | Gene ID | Name | Sequences | Note |
| --- | --- | --- | --- | --- |
| *E. coli* | 945645 | *FabG* | TTGCGTCACGCGGTATTACT | Forward |
|  |  |  | CGCCGTTCACATGCAAAGTT | Reverse |
|  | 947845 | *rpsL* | ACCACGTACGGTGTGGTAAC | Forward |
|  |  |  | ACGTGGCGTATGTACTCGTG | Reverse |
|  |  |  |  |  |
| *P. aeruginosa* | 880433 | *FabG* | GCATTCACGGTAATGGCACG | Forward |
|  |  |  | GACGAGTGGTTCGATGTGGT | Reverse |
|  | 881709 | *rpsL* | CGACCCTGCTTACGGTCTTT | Forward |
|  |  |  | GTGTACGTCTGACCAACGGT | Reverse |
|  |  |  |  |  |
| *A. baumannii* | 31350563 | *FabG* | GAAAGCACGTTTTGGTCGCA | Forward |
|  |  |  | TGTCGCAATAAAACCTGGCG | Reverse |
|  | 31347464 | *rpsL* | ACACGACCACCACGGATAAG | Forward |
|  |  |  | AAGTTCCTGCGTTGAAGGCT | Reverse |
|  |  |  |  |  |
| *K. pneumoniae* | 11847653 | *FabG* | CGTAACCCGCGGGAATTTTG | Forward |
|  |  |  | ACGTCGGGCAAACCAACTAT | Reverse |
|  | 11849946 | *rpsL* | TTTAACGCCGGAGCAGTCAA | Forward |
|  |  |  | GTAAAGTTTGCCGTGTGCGT | Reverse |
