# Supplementary material for: Tachyplesin Causes Membrane Instability That Kills Multidrug-Resistant Bacteria by Inhibiting the 3-Ketoacyl Carrier Protein Reductase FabG
Source: Front Microbiol. 2018 May 1;9:825. doi: 10.3389/fmicb.2018.00825 (PMC5938390; doi:10.3389/fmicb.2018.00825)
Supplement: Supplementary file 2 [file Table_2.DOC]

**Table S2 RNA oligo sequence for RNA interference of *FabG* in *P. aeruginosa***

| **Name** | | **Sequences** | **Note** |
| --- | --- | --- | --- |
| **Negative control** | | UUCUCCGAACGUGUCACGUTT | Forward |
|  |  | ACGUGACACGUUCGGAGAATT | Reverse |
|  |  |  |  |
| **1259** | | GGUAAGGUCGCAUUGGUAATT | Forward |
|  |  | UUACCAAUGCGACCUUACCTT | Reverse |
|  |  |  |  |
| **1534** | | UCUGCUGGUGCGCAUGAAATT | Forward |
|  |  | UUUCAUGCGCACCAGCAGATT | Reverse |
|  | |  |  |
| **1768** | | GCGUGCCAUUACCGUGAAUTT | Forward |
|  |  | AUUCACGGUAAUGGCACGCTT | Reverse |
|  |  |  |  |
| **1430** | | GACGUUUCCAGCGACGAAUTT | Forward |
|  |  | AUUCGUCGCUGGAAACGUCTT | Reverse |
