# Supplementary material for: Tachyplesin Causes Membrane Instability That Kills Multidrug-Resistant Bacteria by Inhibiting the 3-Ketoacyl Carrier Protein Reductase FabG
Source: Front Microbiol. 2018 May 1;9:825. doi: 10.3389/fmicb.2018.00825 (PMC5938390; doi:10.3389/fmicb.2018.00825)
Supplement: Supplementary file 5 [file Image_1.PDF]

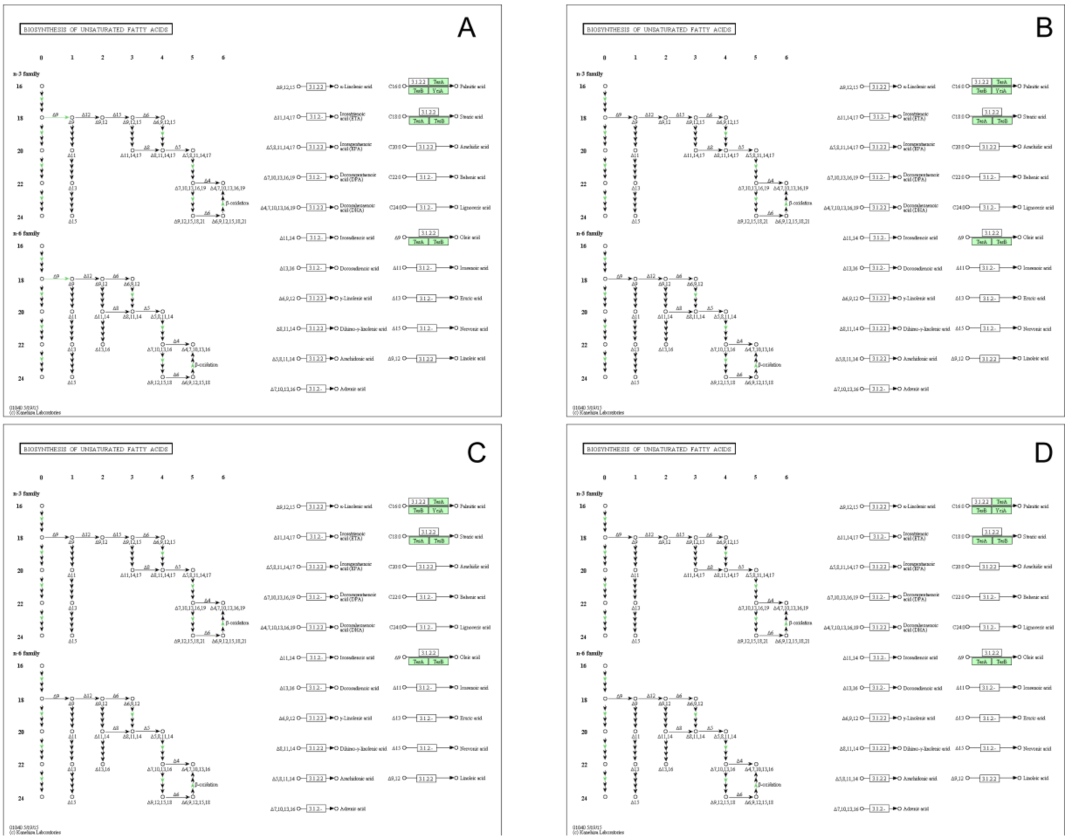

Supplementary Image 1. Kyoto Encyclopedia of Genes and Genomes pathways concerning biosynthesis of unsaturated fatty acids were affected in all the MDR clinical isolates tested.

(A) *E. coli* 513, hyperlink:

http://www.genome.jp/kegg-bin/show_pathway?ecj01040/ecj:Y75_p3334%09red

(B) *A. baumannii* 1408, hyperlink:

http://www.genome.jp/kegg-bin/show_pathway?acb01040/acb:A1S_2061%09red

(C) *K. pneumoniae* 5

http://www.genome.jp/kegg-bin/show_pathway?kps01040/kps:KPNJ2_01584%09red

(D) *P. aeruginosa* 1409

http://www.genome.jp/kegg-bin/show_pathway?pdk01040/pdk:PADK2_09275%09red/pdk:PADK2_25405%09red
